# The soil bacterial community regulates germination of *Plasmodiophora brassicae* resting spores rather than root exudates

**DOI:** 10.1371/journal.ppat.1011175

**Published:** 2023-03-02

**Authors:** Yao Wang, Xiaorong Zheng, Sarenqimuge Sarenqimuge, Andreas von Tiedemann

**Affiliations:** Division of Plant Pathology and Crop Protection, Department of Crop Sciences, Georg August University Göttingen, Göttingen, Germany; University of Cologne, GERMANY

## Abstract

Clubroot, caused by *Plasmodiophora brassicae*, is a severe soil-borne disease that restricts the production of cruciferous crops worldwide. A better understanding of biotic and abiotic factors regulating germination of *P*. *brassicae* resting spores in the soil is significant for developing novel control methods. Previous studies reported that root exudates can trigger *P*. *brassicae* resting spore germination, thus enabling a targeted attack of *P*. *brassicae* on host plant roots. However, we found that native root exudates collected under sterile conditions from host or non-host plants cannot stimulate the germination of sterile spores, indicating that root exudates may not be direct stimulation factors. Instead, our studies demonstrate that soil bacteria are essential for triggering germination. Through 16s rRNA amplicon sequencing analysis, we found that certain carbon sources and nitrate can reshape the initial microbial community to an inducing community leading to the germination of *P*. *brassicae* resting spores. The stimulating communities significantly differed in composition and abundance of bacterial taxa compared to the non-stimulating ones. Several enriched bacterial taxa in stimulating community were significantly correlated with spore germination rates and may be involved as stimulation factors. Based on our findings, a multi-factorial ‘pathobiome’ model comprising abiotic and biotic factors is proposed to represent the putative plant-microbiome-pathogen interactions associated with breaking spore dormancy of *P*. *brassicae* in soil. This study presents novel views on *P*. *brassicae* pathogenicity and lays the foundation for novel sustainable control strategies of clubroot.

## Author summary

Cruciferous crops are crucial in our daily lives as vegetables, sources of oil, green manure and animal feed. Clubroot caused by *Plasmodiophora brassicae* is a severe soil-borne disease that significantly threatens the production of cruciferous crops. The disease is difficult to control and therefore effective sustainable control methods are urgently required. An important step in the life cycle of *P*. *brassicae* is the germination of resting spores which is required for root infection because dormant spores cannot infect plants. Previous studies believed that plant root exudates are the key factors to trigger spore germination. However, our study shows contrasting results and suggests that soil bacteria play an important role in stimulating germination of resting spores. A model is proposed to elucidate the relationship between the plant, the pathogen and biotic and abiotic factors in the soil. These findings provide novel insights into the regulation of dormancy and germination of resting spores of *P*. *brassicae* as key processes preceding infection, which may enable the development of innovative integrated sustainable control strategies. For example, the microbial community could be artificially manipulated (synthetic community) to stimulate spore germination in the absence of host plants in order to reduce soil inoculum. This could significantly enhance the soil sanitation effects of particular crop rotation cycles and lead to durable disease control.

## 1. Introduction

Cruciferous crops are economically important as vegetables, edible and industrial oil sources, animal feeds, manure and biofuel [1]. Cruciferous vegetables are rich in nutrients and also have medical benefits for human health [2,3]. With growing market demands for yield and quality, the global needs for efficient and sustainable cultivation of cruciferous crops are increasing. However, clubroot caused by *Plasmodiophora brassicae* is a severe soil-borne disease that threatens the production of cruciferous crops. The main symptoms of clubroot are the presence of root galls, stunted growth and accelerated flowering [4]. This disease causes significant yield and quality losses (up to 40%), as well as a decrease in land capital value [5].

Due to the severe damage caused by *P*. *brassicae* and its worldwide geographical distribution, various control methods have been developed, such as soil liming [6], chemical treatments [7–9], crop rotation [10], resistant cultivars [11] and biocontrol [12,13]. However, there are no pesticides approved for clubroot control in EU countries. The efficacy of biocontrol in the field is limited [14,15]. Clubroot resistant cultivars of Brassica crops have been available for several years, but resistance in these crops has not been sustainable and several cases of virulent clubroot isolates overcoming the race-specific clubroot resistance have been reported [16,17]. While these limitations pose a significant challenge to effective clubroot control, the lacking knowledge on key processes in the pathogen life cycle still hinders the development of novel disease control strategies.

Resting spores of *P*. *brassicae* can survive in the soil for many years [18]. The germination of resting spores is essential for the pathogenicity of *P*. *brassicae* as it releases primary zoospores which incite root infection. It is generally believed that *P*. *brassicae* spores remain dormant in soil to withstand adverse conditions until dormancy is broken by chemical signals from the compatible host. Previous studies have reported that root exudates of host and non-host plants can stimulate the germination of *P*. *brassicae* resting spores under experimental conditions [19,20]. However, these studies did not clearly explain the stimulatory mechanism, why root exudates from non-host plants can also stimulate resting spore germination and which specific substances in root exudates may induce germination.

Apart from signals from the host plant, a potential role of the soil microbial community in regulating *P*. *brassicae* resting spore dormancy and germination cannot be assumed, taking into account the major impact of soil microbes on the germination of fungal spores, e.g. in suppressive soils [21]. Recent studies have proposed the ‘pathobiome’ concept, which links the pathogenic agent with its surrounding microbial community and considers their interactions as important drivers of pathogenic processes [22,23]. Accordingly, Daval et al., [24] proposed that the soil microbial diversity levels had an impact on the development of clubroot disease. However, the manner in how the soil microbial community may regulate and incite *P*. *brassicae* resting spore germination is still unknown. The aim of the present study therefore was to verify the role of root exudates from host and non-host plants and to explore the impact of further biotic and abiotic factors in the soil involved in the induction of resting spore germination. A better and more comprehensive understanding of the ecological factors associated with key steps in the life cycle of *P*. *brassicae* required for root infection will provide a novel basis to develop innovative strategies for the sustainable control of this notorious pathogen.

## 2. Materials and methods

### 2.1. Preparation of spore suspension

Resting spores were extracted from the root galls of Chinese cabbage, *Brassica rapa* cv. Granaat, inoculated with the single spore isolate H1 (provided by Prof. Elke Diederichsen, Freie Universität Berlin). Briefly, the galls were homogenized with sdH_2_O using an autoclaved homogenizer (Ultra-Turrax T25, IKA-Werke GmbH & Co. KG, Germany) and filtered through eight layers of sterile 50 μm nylon mesh. The filtrate was centrifuged at 500 rpm for 10 min, and subsequently the supernatant was transferred into new tubes and centrifuged for 10 min at 3,500 rpm to obtain the pellet. The pellet was rinsed twice with sdH_2_O. One portion directly resuspended with sdH_2_O was considered non-sterile spore suspension containing bacterial contamination. The other part was surface-sterilized with freshly prepared 2% chloramine-T (Sigma-Aldrich, Germany) (w/v) for 20 min. After rinsing twice, the spores were incubated with antibiotics consisting of 2 μg ml^-1^ colistin sulfate (Sigma-Aldrich, Germany), 2 μg ml^-1^ vancomycin hydrochloride (Sigma-Aldrich, Germany) and 12 μg ml^-1^ cefotaxime sodium (Fluka, Germany) in the dark overnight to obtain the sterile spore suspension. The concentration was adjusted to 1.5×10^8^ spores ml^-1^ using a haemocytometer. The spore viability in both fractions was assessed and confirmed by the CFW-PI dual staining method [25].

### 2.2. Collection of root exudates

Root exudates of rapid cycling rape (*B*. *napus*), perennial ryegrass (*Lolium perenne)* and tomato (*Solanum lycopersicum*) were collected by Petri dish cultivation (PDC) and with a hydrophobic trapping system (HTS). Seeds were surface disinfected with 70% ethanol for 10 min, followed by 1% NaOCl for 10 min, and rinsed three times with sdH_2_O. Surface-disinfected seeds were placed on Murashige and Skoog (MS) basal medium covered with a layer of autoclaved cellophane and pre-germinated in a growth chamber at 25°C with 14 h photoperiod. Contaminated seeds were discarded.

#### 2.2.1. Petri dish cultivation (PDC)

Forty 7-day-old seedlings of each plant species were transferred to a Petri dish containing 20 ml autoclaved 1/10 strength Hoagland solution (Sigma-Aldrich, Germany) or sdH_2_O. The Petri dishes were placed in a sterile plastic container and incubated in the growth chamber at 25°C, 70% humidity and 16h daily light period (irradiance at 180 μE m^−2^ s^−1^). After seven days, the root exudates of each plate were harvested with a syringe and passed through a 0.2 μm sterilization filter. The collected root exudates were used for bioassays immediately.

#### 2.2.2. Hydrophobic trapping system (HTS)

A hydroponic system modified from Tang and Young [26] was established for trapping hydrophobic exudates from undisturbed living roots under sterile conditions. Diluted (1/5 strength) Hoagland solution was continuously circulated through the root system by an air pump (SCHEGO, Schemel & Goetz, Germany) and through two columns with XAD8 resin and XAD4 resin (Amberlite, Sigma-Aldrich, Germany). Extracellular hydrophobic metabolites were selectively adsorbed by the resin, while inorganic nutrients were recycled to sustain plant growth (S1 Fig). Additional Hoagland solution was supplied as needed during the whole cultivation period. The whole system was sterilized and run under sterile conditions in an ozone disinfected climate chamber at 23/18°C (day/night), 70% humidity, and 14 h daytime. One system without plants served as blank control.

To obtain sterile conditions, all non-living materials were autoclaved and the climate chamber was disinfected with ozone (fumigation with 10 ppm ozone for 5 h). Glass containers (5L, O.D. × H 18.6 cm × 33.5 cm) were filled up with perlite and all the components were wrapped with aluminum foil and autoclaved twice at 121°C for 20 min. One milliliter sterile pipette tips were cut at the bottom and filled with 800 μl of 1% water agar. Seeds with radicles pre-germinated on MS plate were placed on the surface of water agar in the tips. Twenty-two tips with each plant species were inserted into a plastic membrane with micro-porous filter strips covered on top of the container.

The liquid chromatography columns (Luer Lock, Non-jacketed, bed volume 8 ml, I.D. × L 1.0 cm × 10 cm) (Sigma-Aldrich, Germany) and the stopcocks were washed with sdH_2_O and soaked in 75% ethanol until use. Amberlite XAD4 resin and XAD8 resin were rinsed with sdH_2_O, followed by HPLC grade methanol. The cleaned resin was stored in methanol at 4°C in the dark. Columns were packed with 6 ml XAD4 resin or 8 ml XAD8 resin, and then rinsed with sdH_2_O to remove methanol. Root exudates were separately collected from different plant species at three growth stages (BBCH 14, 52 and 64) by XAD4 and XAD8 resin columns. At different growth stages, one XAD8 resin column followed by one XAD4 resin column was connected to the system to absorb the root metabolites. After two days of absorption, the columns were taken off and pre-washed with 40 ml of gradient (5%-75%) methanol. After washing, the columns were eluted with 80 ml HPLC grade methanol. The eluate was stored at -20°C until use.

### 2.3. Root exudates bioassay

The eluted root exudates from XAD4 and XAD8 resin were dried in a speed vacuum concentrator at 30°C. The residue was dissolved with 1/10 strength Hoagland solution or sdH_2_O. To examine the effects of root exudates, 100 μl surface-disinfected (sterile) spore suspension was incubated with 1 ml of each root exudate preparation from PDC and HTS in 2 ml Eppendorf tubes at 25°C in the dark. Hoagland solution or sdH_2_O mixed with spores were used as controls. At least 100 resting spores of each sample were examined daily under the differential interference contrast microscope. Resting spores were considered to have germinated if they appeared empty [25].

### 2.4. Soil suspension bioassay

Rhizosphere and bulk soil samples were collected from an oilseed rape (cv. Bender) field in Weende, Goettingen, Germany (51°33’48.6"N 9°56’47.7"E). Thirty grams of soil were thoroughly soaked with 40 ml sdH_2_O for 1 h. The slurry was passed through sterile filter paper to obtain a non-sterile soil suspension. Part of the filtrate was subjected to a 0.2 μm sterilization filter to obtain a sterile soil suspension. An aliquot of 100 μl non-sterile spore suspension was incubated with 1 ml non-sterile or sterile soil suspension at 25°C. Hoagland solution or sdH_2_O mixed with spores served as control. The germination rate of resting spores was checked daily.

### 2.5. Soil moisture bioassay

The field soil was autoclaved at 121°C for 20 min to exclude the effect of viable microbes. Twenty-two grams of autoclaved and non-autoclaved soil was filled in multiple trays. *P*. *brassicae* spore suspension (5 ml 7×10^7^ spores ml^-1^) was inoculated into the soil and mixed well. The soil was treated with different amounts of sdH_2_O to obtain different moisture levels of 6%, 50% and 90%. The samples were incubated in a climate chamber at 25°C for three weeks. The resting spores in the soil were checked under the microscope to calculate the germination rates.

### 2.6. Bacterial suspension and bacterial filtrate bioassay

The bacterial strains Iso4, A4, *Bacillus subtilis* JH642 (obtained from Prof. Dr. Dieter Jahn, TU Braunschweig) and *Escherichia coli* GSPB48 (Göttingen Collection of Phytopathogenic Bacteria, Germany) were cultured in Luria-Bertani (LB) medium at 37°C overnight and centrifuged at 5,000 rpm for 10 min to remove the medium. The bacterial cells were resuspended with sdH_2_O and adjusted to an OD_600_ of ca. 0.2. A portion of the bacterial suspension was passed through a 0.2 μm sterilization filter to obtain the filtrate. A volume of 100 μl of 0.9 × 10^8^ spores ml^-1^ non-sterile (or sterile) spore suspension and 100 μl bacterial suspension or filtrate were incubated in 1 ml Hoagland solution or sdH_2_O. Resting spores incubated with 1/10 strength Hoagland solution or sdH_2_O alone were used as controls. The germination rates of resting spores were checked after 7 days of incubation.

### 2.7. Inorganic compounds bioassays

Several inorganic compounds contained in the Hoagland product formulation were tested. Concentration of inorganic compounds used for bioassays is based on full strength or 1/10 strength Hoagland solution (S1 Table). Non-sterile spores were incubated in 1 ml of different inorganic solutions with 100 μl of Iso4 suspension (OD_600_ = 0.326) at 25°C in the dark. The spores mixed with inorganic solutions alone were also compared. 1/10 strength Hoagland and sdH_2_O were used as controls. The spore germination rates were checked at 7 days post-incubation under the microscope.

### 2.8. ^15^N isotope analysis

^15^N-enriched potassium nitrate (0.6 mM; 10 atom% K^15^NO_3_, Sigma-Aldrich) was used to examine the role of nitrate in stimulating spore germination. Two milliliters of non-sterile spore suspension with or without 3 ml of *E*. *coli* suspension was incubated in 20 ml K^15^NO_3_ at 25°C. Resting spores with or without *E*. *coli* were cultured in sdH_2_O without labelling as control. After 0, 3 and 7days of incubation, the resting spores and *E*. *coli* were separated by 16% Ficoll 400 (w/v) (Carl Roth GmbH & Co. KG, Germany) at 1,850 rpm for 15 min twice. The separated resting spores were rinsed with sdH_2_O four times and incubated in the oven at 50°C overnight for isotope analysis. The total N and total ^15^N abundances of each sample were measured using elemental analyser-isotope-ratio mass spectrometry (EA-IRMS) at the Centre for Stable Isotope Research and Analysis (University of Göttingen, Germany).

### 2.9. Bacterial community analysis

#### 2.9.1. Sample preparation

*B*. *subtilis* JH642 bacterial suspension and isolate A4 bacterial filtrate were used as carbon sources. Hoagland solution (1/10 strength) and 6 mM KNO_3_ were used as nitrogen sources. For the treatments (Table 1), 2 ml of spore suspension and 2 ml of carbon source were incubated in 20 ml nitrogen source at 25°C for 7days. The germination rates were determined, when the samples were harvested. The non-sterile spore suspension collected at day 0 post-incubation represented the initial microbial community originated from the root galls.

**Table 1:** The treatments used for bacterial community analysis.

Treatment	Spores	Carbon source	Nitrogen source	Incubation time
HB	Non-sterile	*B*. *subtilis* suspension	Hoagland	7days
KB	Non-sterile	*B*. *subtilis* suspension	KNO_3_	7days
A4f	Non-sterile	A4 filtrate	Hoagland	7days
H	Non-sterile	None	Hoagland	7days
sd	Non-sterile	None	sdH_2_O	7days
NS	Non-sterile	None	sdH_2_O	0days
SS	Sterile	None	sdH_2_O	0days

#### 2.9.2. DNA extraction

The above samples were spun at 10,000 rpm to pellet the cells and sniped in liquid nitrogen. Samples were lyophilized and crushed with 4 mm beads to powder for DNA extraction. Total DNA was extracted by the CTAB method [27] with autoclaved reagents and resuspended in 50 μl of sterile Tris-EDTA buffer. The extracted total DNA stained with Midori green (Nippon Genetics Europe GmbH, Germany) was qualified and quantified with 1% agarose gel electrophoresis. DNA of *E*. *coli* strain GSPB48 was extracted and used as positive templates. Empty tubes were extracted in parallel and used as non-template controls (NTCs) to assess the presence of contaminants.

#### 2.9.3. 16S rRNA gene amplification and sequencing

The V3 and V4 regions of the 16S ribosomal RNA gene were amplified using a pair of primers D-Bact-0341-b-S-17: 5’-TCGTCGGCAGCGTCAGATGTGTATAAGAGACAGCCTACGGGNGGCWGCAG-3’ and D-Bact-0785-a-A-21: 5’-GTCTCGTGGGCTCGGAGATGTGTATAAGAGACAGGACTACHVGGGTATCTAATCC-3’ [28] that included Illumina adapter overhang nucleotide sequences and the gene-specific sequences. The amplification reaction was carried out in 50 μl volumes that consisted of 10 μl 5×Phusion GC Buffer, 10 μM primer of each, 0.2 μl of 50 mM MgCl_2_, 2.5 μl DMSO, 1 μl of 10mM dNTPs (Thermo Fisher Scientific, USA), 1 Unit Phusion High Fidelity DNA Polymerase (Thermo Fisher Scientific, USA) and 25 ng DNA template. PCR was performed using the following program: initial denaturation at 98°C for 1 min, followed by 25 cycles consisting of denaturation at 98°C for 45 s, annealing at 55°C for 45 s and extension at 72°C for 30 s and a final extension step at 72°C for 5 min. Amplified PCR products of each sample were verified by running the samples on 1% agarose gel (Roth, Germany) with FastGene 100 bp DNA ladder. The PCR products with the appropriate size (~550bp) were individually purified using bead-cleanup (SPRIselect, Beckman Coulter, USA). The amplicons were quantified using the Qubit Fluorimeter (Invitrogen, Carlsbad, USA) twice and placed in a 96-well plate. A second PCR was performed to add barcodes to the amplicons. The amplicons were pooled and sequenced with MiSeq instrument and v3 chemistry (Illumina, San Diego, USA) using the dual index paired-end approach (2×300 bp) at Göttingen Genomics Laboratory. The raw sequencing data for 16S rRNA gene sequencing results have been deposited at the Sequence Read Archive under accession numbers PRJNA884423 (https://www.ncbi.nlm.nih.gov/sra/PRJNA884423).

#### 2.9.4. Sequence data analysis

The raw data was processed according to the description by Liu et al. [29] using the default settings. Briefly, the sequences were processed by USEARCH as following: merging pair-end reads and relabeling names; cutting barcodes and primers; filtering of low-quality reads and removal of redundancy. The filtered reads were denoised by UNOISE3 and the representative sequences were picked by VSEARCH at 97% similarity. ASVs (amplicon sequence variants) were aligned to RDP database (rdp_16s_v18) to remove plastids and non-bacteria. An abundance table was generated by USEARCH. The bacterial community samples were separated into three groups, associated with either ‘high’ or ‘low’ germination rates of resting spores, compared with the initial community. To identify features differentially represented between the groups, differentially abundant taxa were selected using the LEfSe (linear discriminant analysis, LDA) effect size [30]. Differential abundance of taxa between the groups was tested by Wilcoxon rank-sum test or Welch’s t-test, *P*-value was corrected as false discovery rate (FDR). Functional annotations were carried out using FAPROTAX (Functional Annotation of Prokaryotic Taxa) v.1.2.4 and visualized by STAMP (statistical analysis of taxonomic and functional profiles) [31]. The correlation between the resting spore germination rates and the relative abundance of bacteria was calculated by Spearman’s correlation coefficient. To understand the correlations among different genera, co-occurrence network was constructed based on the 16S rRNA gene amplicon data. The bacterial correlations in the high and low germination rate groups were analyzed using Spearman’s correlation coefficient constructed with the “WGCNA” package. The significantly correlated genera (ρ > 0.8 or < -0.8, *P* < 0.01) were visualized using the software Gephi version (0.9.3).

### 2.10. Sugar and amino acid bioassays

Different 50 mM solutions of sugars (glucose, sucrose, trehalose, maltose, fructose and xylose) and L-amino acids (alanine (Ala), arginine (Arg), asparagine (Asn), aspartic acid (Asp), cysteine (Cys), glutamic acid (Glu), glutamine (Gln), glycine (Gly), histidine (His), isoleucine (Ile), leucine (Leu), lysine (Lys), methionine (Met), phenylalanine (Phe), proline (Pro), serine (Ser), threonine (Thr), tryptophan (Trp), and valine (Val)) were tested as carbon sources to check the effects on the germination of resting spores. For the bioassay, 100 μl sterile/non-sterile spores were incubated in 500 μl different carbon sources mixed with 500 μl of 50 mM KNO_3_ at 25°C in the dark. Treatments without KNO_3_ were also included to evaluate the effects of carbon sources alone. The germination rate of resting spores was examined after 14 days of incubation under the microscope.

### 2.11. Statistical analysis

Statistical analyses were carried out with SPSS (version 26, IBM) and R (version 4.0.2). A mixed-model analysis of variance (ANOVA) was performed using a general linear model (GLM) to determine significance levels for main factors and interactions between factors. Subsequently, Tukey’s Multiple Comparisons Tests were conducted to determine the statistically significant differences among means. All bioassays were repeated at least three times.

## 3. Results

### 3.1. Effect of root exudates on resting spore germination

The viability of non-sterile and sterile resting spores which was assessed by CFW-PI dual staining before the bioassays was about 98%. Root exudates collected from PDC and HTS were mixed with sterile spores to examine their effects on stimulating resting spore germination, respectively. The results showed that the germination rates of sterile spores incubated with root exudates based Hoagland solution or sdH_2_O collected from PDC was approximately zero percent. Similarly, root exudates from HTS resuspended in sdH_2_O or Hoagland solution did not induce germination of sterile spores (S2 Table). There were no significant differences in the germination rates of resting spores incubated with root exudates collected from XAD8 resin and XAD4 resin columns, or with root exudates obtained from different plant species and different growth stages.

### 3.2. Effect of soil microbes on resting spore germination

**Fig 1:**
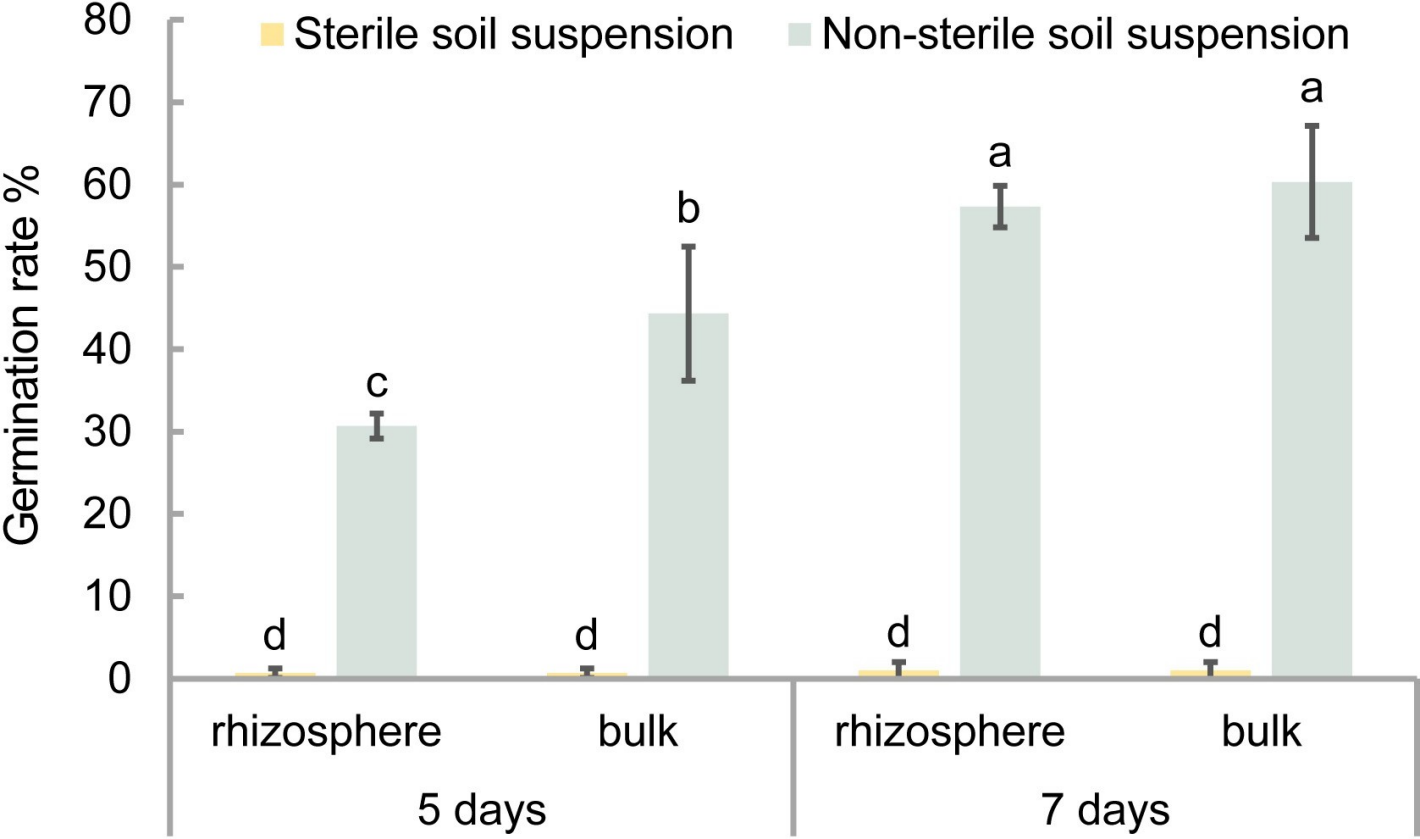
Germination rates of *P*. *brassicae* resting spores incubated in non-sterile and sterile soil suspensions from an oilseed rape field after 5 and 7 days of incubation. Rhizosphere soil was collected from the roots of field-grown oilseed rape cv. Bender at BBCH 18 (eight-leaf stage). Bulk soil was collected from a neighbouring field without plants. Error bars represent standard deviations. Different letters represent significant differences among the treatments (Tukey test. *P* < 0.05, n = 3).

Non-sterile spores incubated with non-sterile or sterile soil suspensions from oilseed rape field soil displayed different germination rates (Fig 1). Both rhizosphere and bulk soil suspensions had similar stimulating effects on spore germination. Resting spores incubated in sterile soil suspensions of both rhizosphere and bulk soil barely germinated up to 7 days post-incubation. However, resting spores incubated in non-sterile soil suspensions had significantly elevated germination rates compared to the sterile soil suspensions, indicating that the soil bacteria are important for stimulating the resting spore germination.

### 3.3. Effect of soil moisture on resting spore germination

Germinated spores were distinguishable from non-germinated spores in soil, when samples had been diluted to facilitate the observation (S2 Fig). Resting spores incubated in autoclaved and non-autoclaved soil with different levels of water holding capacity, showed that germination rates significantly increased with the soil moisture level (Table 2). Resting spore germination rates were dramatically higher in non-autoclaved than in autoclaved soil, when soil moisture was greater than 50%. This indicates that soil moisture is an essential environmental factor for enabling resting spore germination.

**Table 2:** Germination rates of *P*. *brassicae* resting spores incubated in autoclaved and non-autoclaved soil with different levels of moisture.

Soil moisture	Germination rate %	
	Autoclaved soil	Non-autoclaved soil
6%	0.0±0.0 a	0.0±0.0 a
50%	2.0±1.0 a	28.3±3.1 c
90%	12.3±5.0 b	70.6±2.1 d

Value = mean ± SD; Different letters indicate significant differences among all samples (Tukey test, *P* < 0.05)

### 3.4. Effect of bacterial suspensions and filtrates on resting spore germination

**Fig 2:**
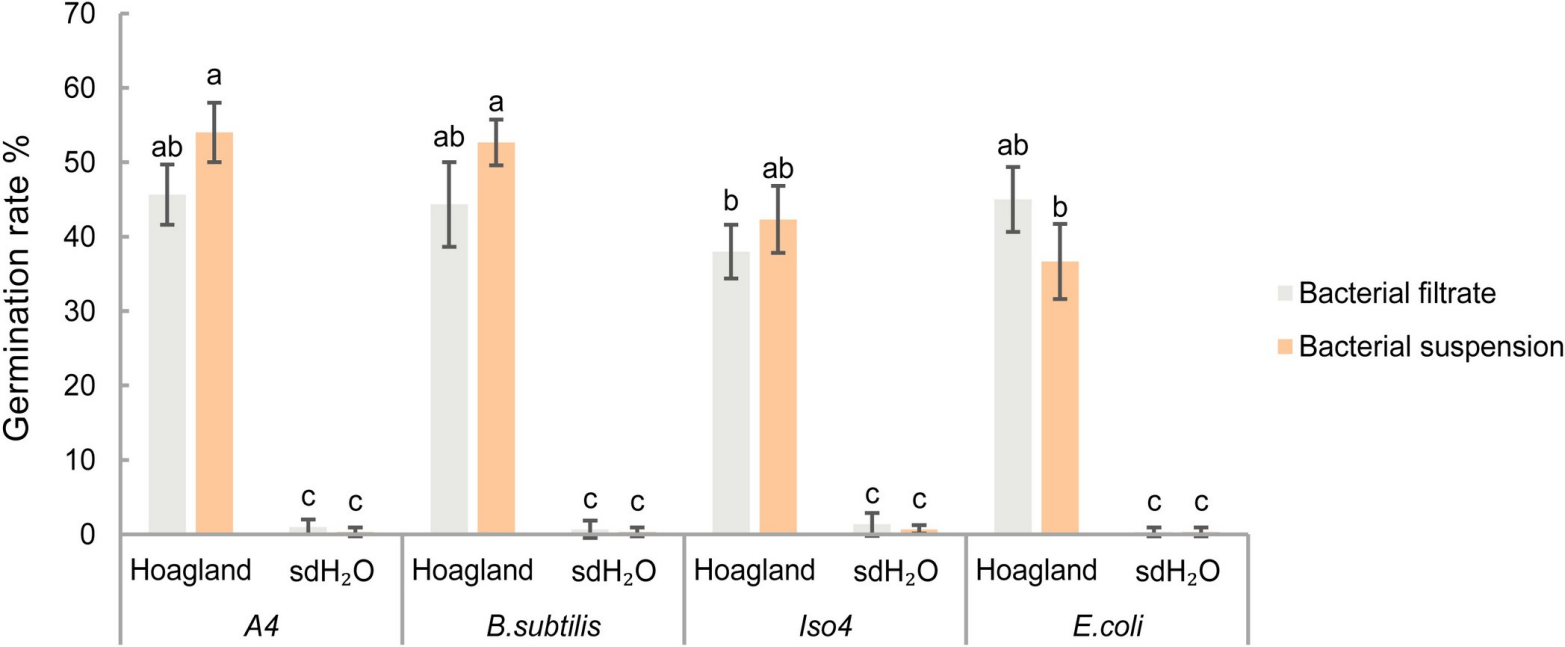
Germination rates of non-sterile resting spores of *P*. *brassicae* incubated with different bacterial suspensions or filtrate with Hoagland solution or sdH_2_O after 7 days. Error bars represent standard deviations. Different letters represent significant differences among the treatments (Tukey test. *P* < 0.05, n = 3).

Without the presence of Hoagland solution, bacterial suspension or filtrate did not induce resting spore germination, indicating the importance of nutrients from Hoagland solution for the germination of non-sterile resting spores. Fig 2 also shows that there were no significant differences in the germination rates of resting spores incubated with bacterial suspensions and filtrates with Hoagland solution, indicating the tested bacteria cells are not essential and they are not the direct stimulants. Sterile spores barely germinated in all treatments, even with bacterial suspension or filtrate in Hoagland solution (S3 Table). This indicates the microbial contaminations in the non-sterile spore suspension are important for the germination of *P*. *brassicae* resting spores.

### 3.5. Effect of inorganic compounds on resting spore germination

**Fig 3:**
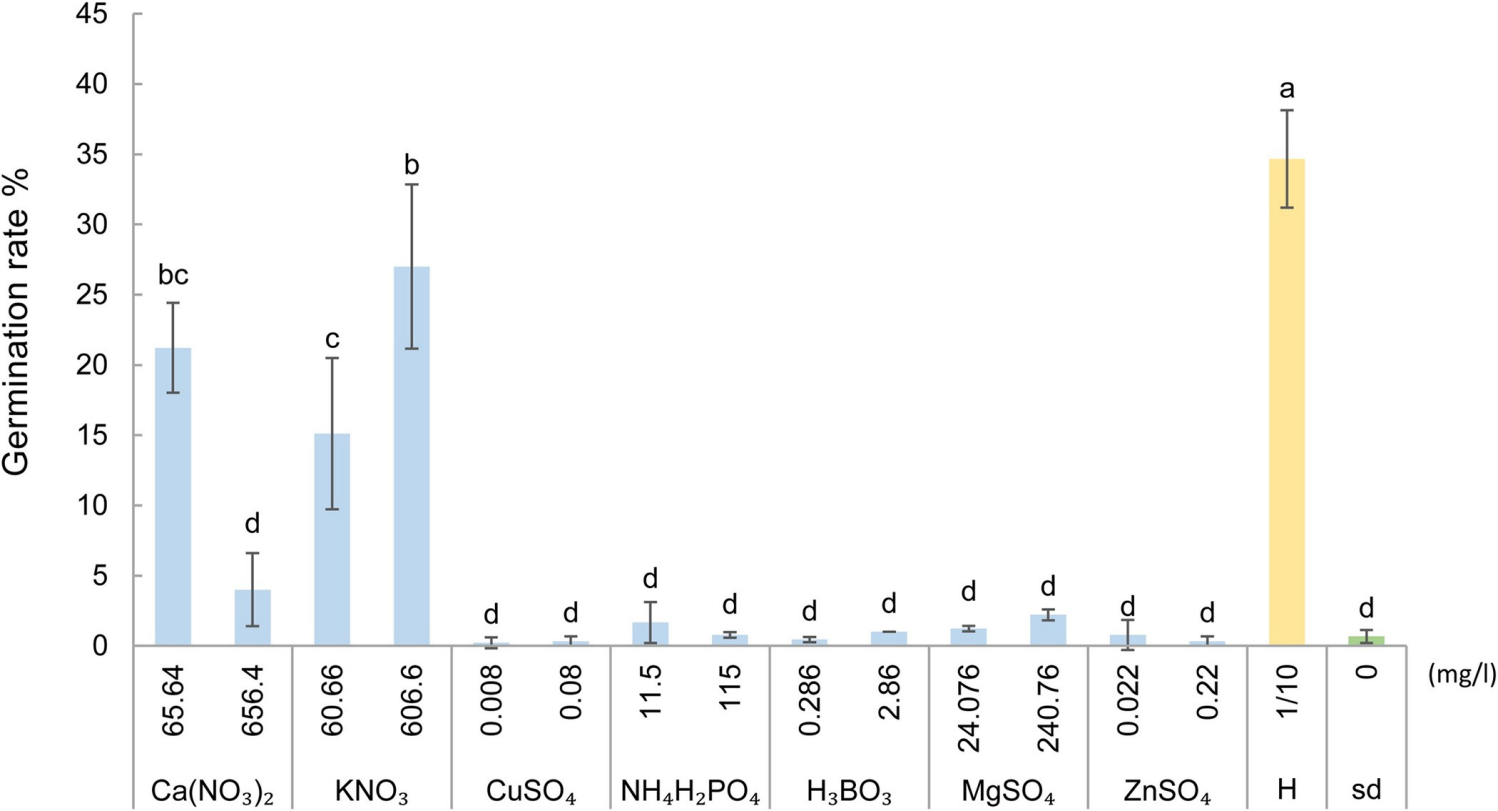
Germination rates of non-sterile resting spores of *P*. *brassicae* incubated with Iso4 bacterial suspension in various solutions after 7 days. The positive control of 1/10 strength Hoagland solution (H) is indicated in yellow. The negative control of sdH_2_O (sd) is indicated in green. Error bars represent standard deviation s. Different letters represent significant differences among the treatments (Wilcoxon rank sum test. *P* < 0.05, n = 3).

Germination rates of non-sterile resting spores cultured in various inorganic solutions without bacterial suspension were almost 0%. The germination rates of non-sterile spores strongly increased in the presence of particular nutrient compounds and Iso4 bacterial suspension (Fig 3). The germination rate of the negative control (sdH_2_O) was close to 0%, and the positive control, 1/10 strength Hoagland solution, had the highest germination rate of 34.6%. Calcium nitrate and potassium nitrate significantly triggered germination rates, while the other nutrient compounds did not differ from the negative control. The higher concentration of potassium nitrate (606.6 mg/l) resulted in a higher germination rate of 27%, while conversely, the elevated concentration of calcium nitrate (656.4 mg/l) resulted in a lower germination rate of 4%. This indicates that nitrate appears to be essential for resting spore germination, while the cations differentially affect germination rates.

### 3.6. Role of nitrate in triggering spore germination

**Fig 4:**
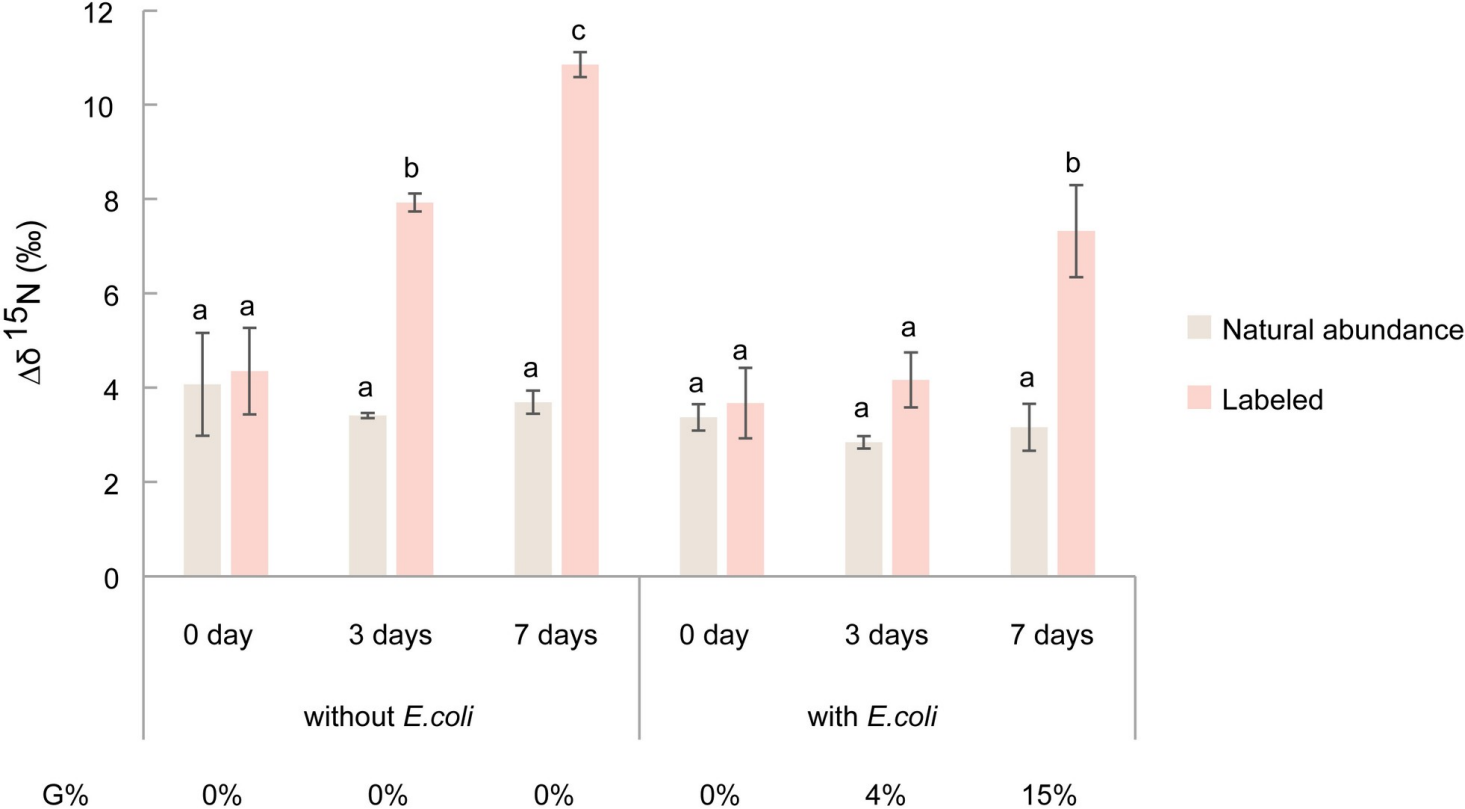
^15^N enrichment (Δδ^15^N) of *P*. *brassicae* resting spores incubated with potassium nitrate with (+ *E*. *coli*) or without (- *E*. *coli*) the presence of *E*. *coli* suspension and corresponding germination rates (G%) after different incubation times. Error bars indicate standard deviations. Different letters represent significant differences among the treatments (Tukey test. *P* < 0.05, n = 3).

The ^15^N content in resting spores was measured after incubation with unlabeled or 10% ^15^N-enriched potassium nitrate and with or without addition of *E*. *coli* suspension. The nitrogen content of resting spores was about 4.5% on average. The results showed that ^15^N enrichment in resting spores increased with incubation time regardless of the presence of additional *E*. *coli* suspension compared to natural abundance (Fig 4). There was no relationship between the enrichment of ^15^N and the germination rates of resting spores, indicating that nitrate is not stimulating germination by directly acting as a nutrient taken up by resting spores.

### 3.7. Bacterial community analysis

**Fig 5:**
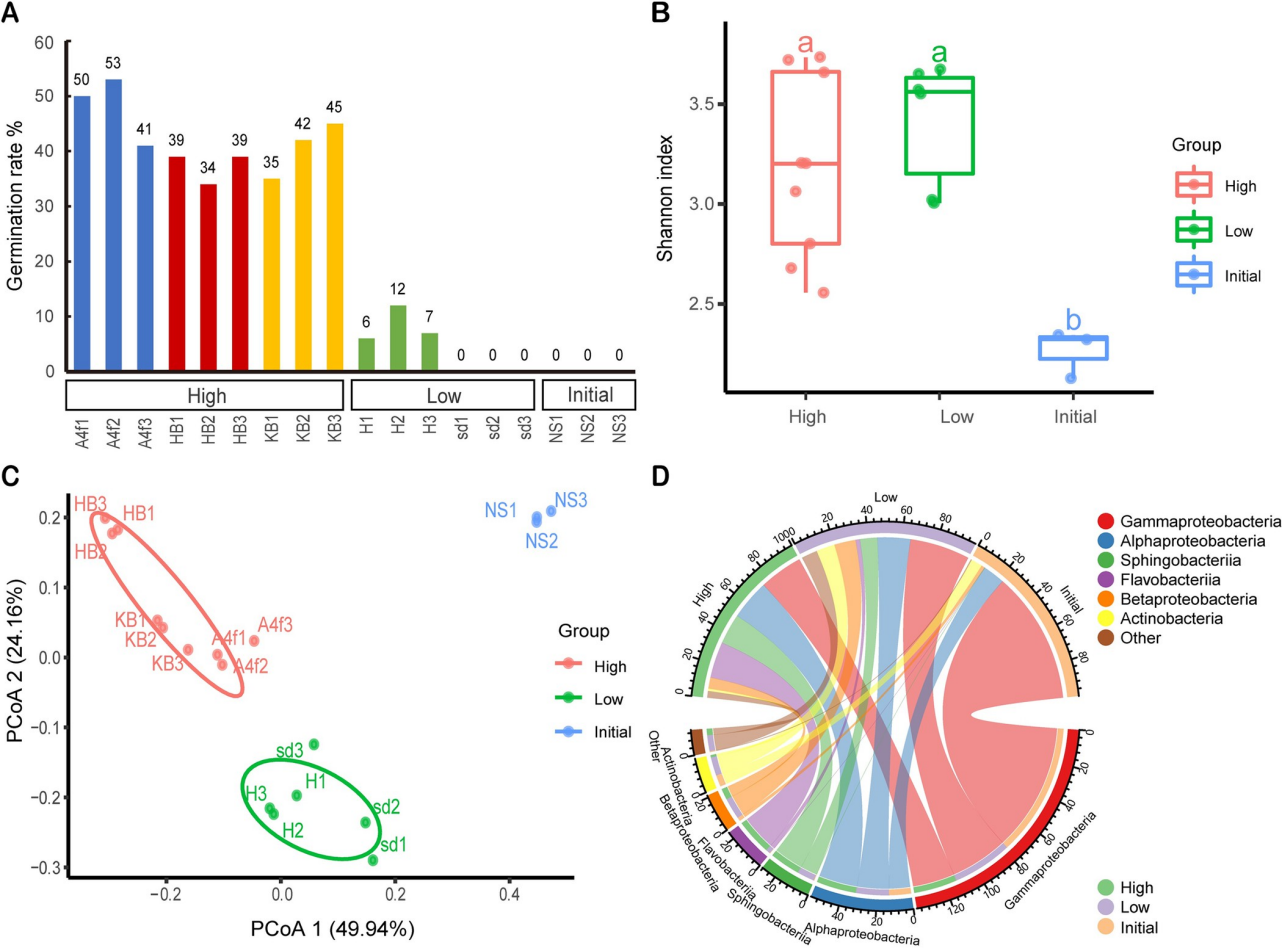
The shift of bacterial community composition after 7 days of incubation. Comparison of bacterial communities differently treated and inducing ‘high’ or ‘low’ germination rates of *P*. *brassicae*, in relation to the ‘initial’ untreated non-sterile resting spores. (A) Germination rate of resting spores of *P*. *brassicae* in each sample used for 16S rRNA gene amplicon sequencing. (B) Shannon index of the microbiota from ‘high’, ‘low’ and ‘initial’ groups. (C) Principal coordinate analysis (PCoA) based on the Unifrac distance showing that the bacteria of ‘high’ germination rate group separate from those of ‘low’ germination and the initial groups (*P* < 0.001, PERMANOVA by Adonis). (D) Chord diagram visualizing community structure in different groups at class-level. HB: *B*. *subtilis* cell suspension with 1/10 strength Hoagland solution; KB: *B*. *subtilis* cell suspension with 6 mM potassium nitrate; A4f: filtrate of bacterial strain A4 with 1/10 strength Hoagland solution; sd: sterilized deionized water; H: 1/10 strength Hoagland solution; NS: non-sterile resting spore suspension (initial bacterial community).

In the presence of soil bacteria, the germination of resting spores was stimulated after 7 days of incubation showing different germination rates in each treatment (Fig 5A). Treatments with bacterial filtrate/bacterial suspension and Hoagland solution/potassium nitrate (A4f, HB and KB) resulted in strongly enhanced germination rates compared to treatments with either Hoagland (H) or sdH_2_O (sd) alone. Accordingly, hereafter, treatments and bacterial communities are designated ‘high’ or ‘low’ when triggering high or low germination rates, respectively, and compared with ‘initial’ bacterial communities associated with non-sterile spore suspension.

After 16s rRNA gene amplicon sequencing, we obtained 620,712 reads (average, 34,484; range, 17,514–79,518 reads per sample) from 18 samples after cleaning and filtering (S1A File). The reads were analyzed with USEARCH, removing chimeric and organelle sequences, to obtain 341 ASVs (S1B File). We found that the composition of the bacterial community shifted with different treatments and this was related to the stimulation of resting spore germination. The Shannon index, which accounts for both richness and evenness of species present, showed significant increases in samples after incubation compared to the initial samples (Fig 5B). A Principal Coordinates Analysis (PCoA) was performed to analyze the extent of similarity of the bacterial communities among the three germination groups based on UniFrac distance metrics, indicated that the bacterial composition of the three groups was significantly different (Fig 5C). A chord diagram displayed clear alterations of microbial profiles at class level in the ‘high’ and ‘low’ germination groups compared to the original community in the spore suspension (Fig 5D). The ‘initial’ bacterial communities were dominated by *Gammaproteobacteria*. After 7 days of incubation, the relative abundance of *Gammaproteobacteria* in each sample decreased, while the portion of *Sphingobacteriia* and *Flavobacteriia* increased, especially in the ‘high’ germination group. These changes of relative abundance in each community resulted in different patterns, which were associated with the spore germination rates.

**Fig 6:**
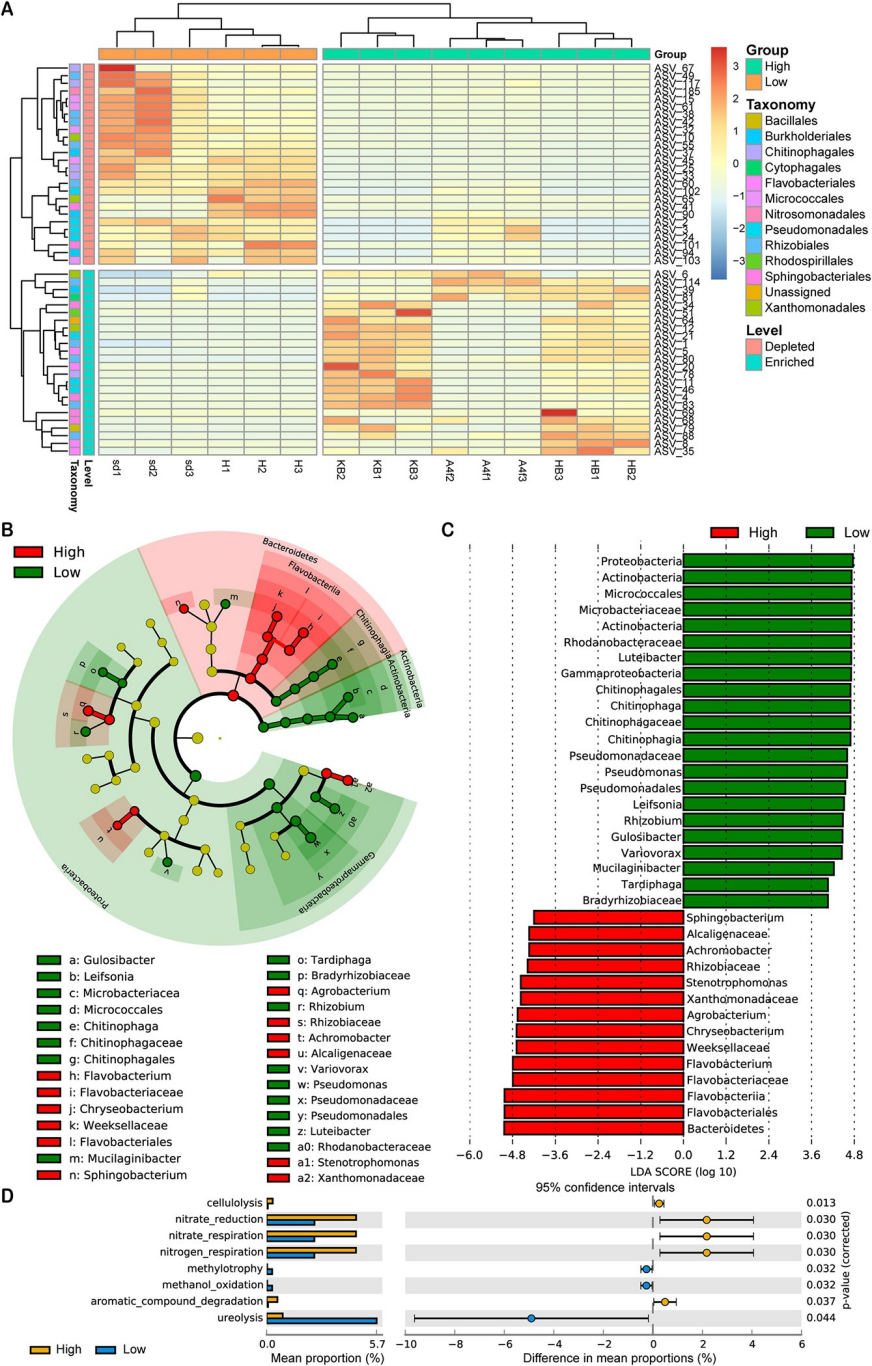
Taxonomic and functional characteristics of differential bacteria in ‘high’ and ‘low’ germination rate groups. (A) Relative abundance of the corresponding ASVs enriched in ‘high’ and ‘low’ germination rate groups (Wilcoxon rank sum test, FDR adjusted *P* < 0.05). (B) Cladograms generated by LEfSe indicating differences in the bacterial taxa between ‘high’ and ‘low’ germination groups. Red bars indicate taxa enriched in ‘high’ germination rate group, green bars indicate taxa enriched in ‘low’ germination group. (C) LDA scores for the bacterial taxa differentially abundant in ‘high’ and ‘low’ groups. Red bars indicate taxa enriched in high germination rate group, and green bars indicate taxa in low germination group. (D) Metabolic and ecological functions of ASVs enriched in ‘high’ and ‘low’ groups based on FAPROTAX (Welch’s t-test, *P* < 0.05).

To further explore the stimulation effect of the microbial community on resting spore germination, we compared the microbial composition between ‘high’ and ‘low’ germination rate groups. According to the bacterial community profiles at the ASV level, the hierarchical heatmap indicated that the 51 most significantly different ASVs detected in all samples showed different patterns in the ‘high’ and ‘low’ germination rate groups (Fig 6A). The cladogram generated by LEfSe indicated differences in the phylogenetic distributions of bacteria in the ‘high’ and ‘low’ germination rate groups. Red color representing *Bacteroidetes* was significantly more enriched in the ‘high’ germination rate group, while green color characterizing *Proteobacteria* prevailed in the ‘low’ germination rate group (Fig 6B).

The linear discriminant analysis (LDA) distribution diagram analysis showed clear differences in the microbiota between the two groups. The genera *Sphingobacterium*, *Achromobacter*, *Stenotrophomonas*, *Agrobacterium*, *Chryseobacterium* and *Flavobacterium* were more abundant in the ‘high’ germination group, while the genera *Chitinophaga*, *Luteibacter*, *Pseudomonas*, *Gulosibacter*, *Leifsonia*, *Rhizobium*, *Variovorax*, *Mucilaginibacter* and *Tardiphaga* were more abundant in the ‘low’ germination group (**Fig 6C**). Moreover, we annotated bacterial functions using FAPROTAX (S2 File), a database for predicting ecologically relevant functions based on current literature [32]. Notably, ASVs especially enriched in the high germination rate group had functions related to nitrate reduction, nitrate respiration and nitrogen respiration (Fig 6D).

**Fig 7:**
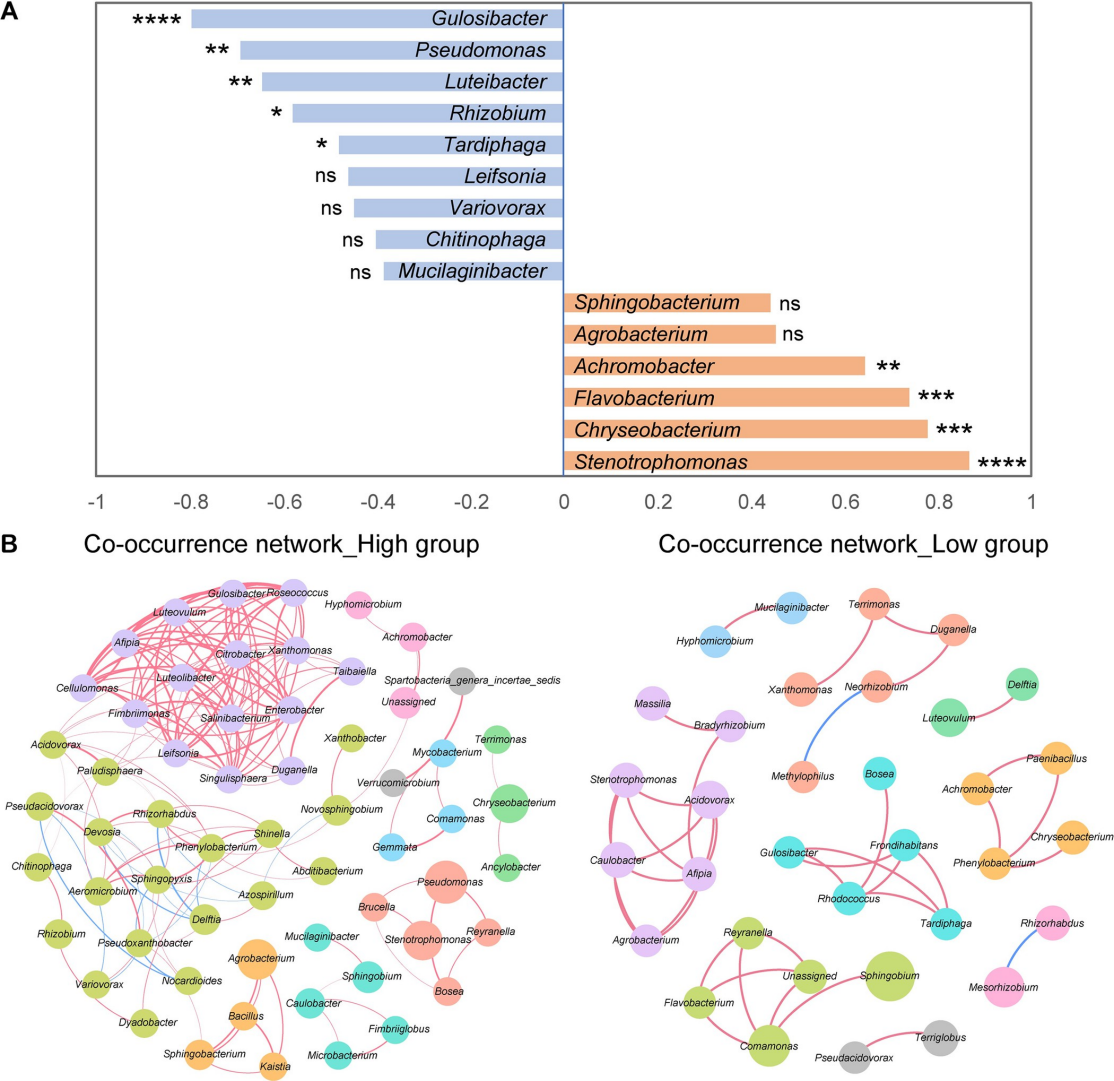
Bacterial genera related to the stimulation of *P*. *brassicae* resting spore germination. (A) Correlation between germination rates and relative abundance of bacteria (Spearman’s ρ, *P* < 0.0001 ****; *P* < 0.001 ***; *P* < 0.01 **; *P* < 0.05 *; ns, not significant). (B) Co-occurrence network of ‘high’ and ‘low’ germination rate groups based on the Spearman correlation algorithms. Each node represents a genus and is colored by modularity. The node size indicates the relative abundance of each genus per group, and the thickness of the line represents the Spearman coefficient (R_s_ > 0.8 or < -0.8, *P* < 0.01). Red links stand for positive, and blue links for negative correlations between nodes.

To further clarify which bacteria are associated with the stimulation of resting spore germination, the enriched bacterial genera identified by LDA were selected to calculate correlations between the relative abundance of bacteria and germination rates. As a result, the relative abundance of *Stenotrophomonas*, *Chryseobacterium*, *Flavobacterium* and *Achromobacter* was clearly positively correlated with spore germination rates (Fig 7A). These correlations were strongly significant with an R-value of 0.87, 0.78, 0.74 and 0.65, respectively. These results suggest that key bacteria, which are able to stimulate spore germination may belong to these genera. We further constructed co-occurrence networks based on genus level from each group using significant Spearman correlations, in order to describe potential relationships occurring among bacteria within the microbial communities. As shown in Fig 7B, the constructed networks of high and low germination rate groups consisted of 60 nodes connected by 172 edges and 34 nodes connected by 38 edges, respectively (S4 Table). This indicates that microbial community of the high germination rate samples featured a more complicated network. The composition of each network differed strikingly with 23 common nodes and there were no shared edges between the two networks.

### 3.8. Effect of sugars and amino acids on resting spore germination

**Fig 8:**
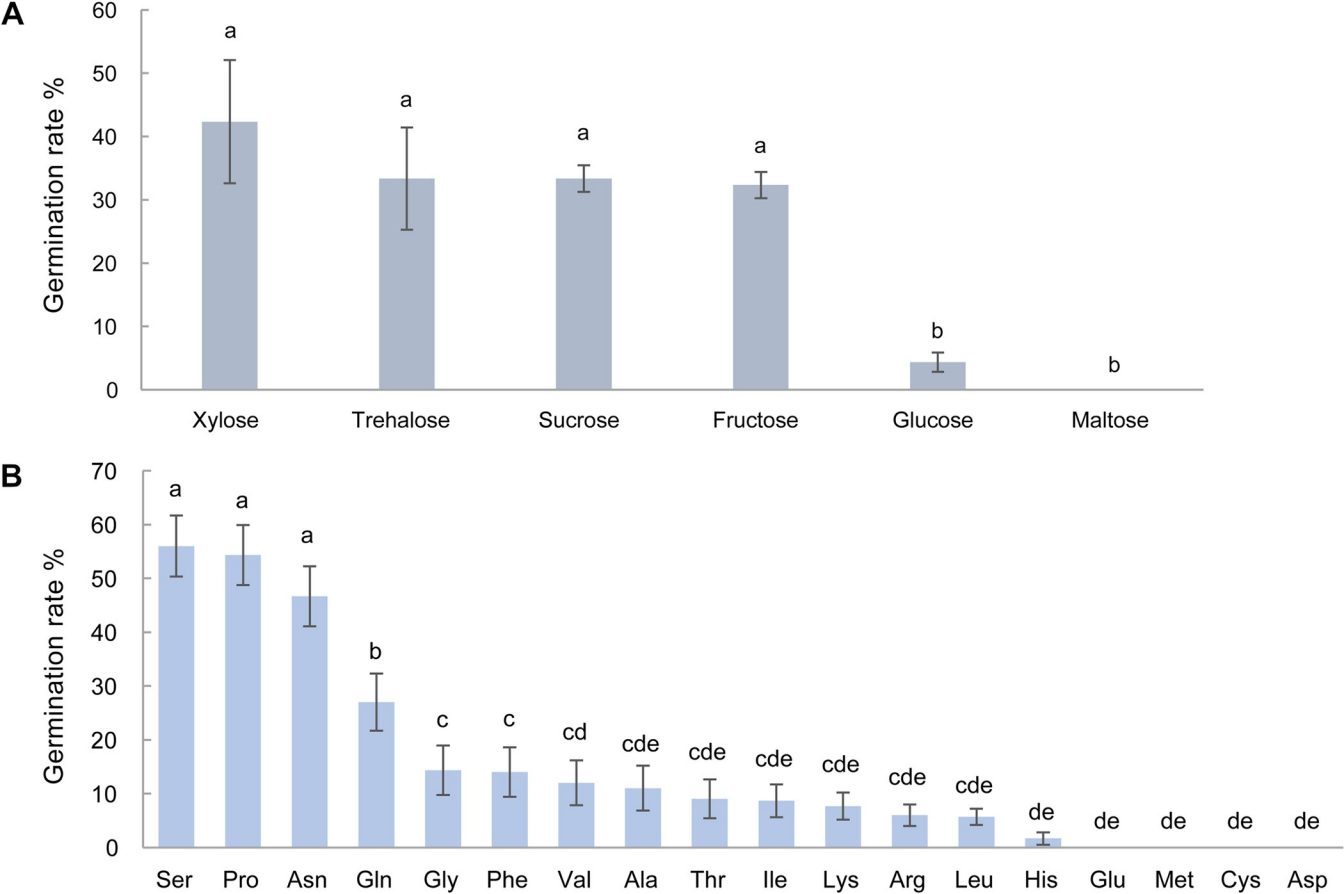
**Germination rates of *P*. *brassicae* resting spores incubated with various sugars (A) and L-amino acids (B) in the presence of nitrate.** Non-sterile spores were incubated with 50 mM potassium nitrate for 14 days. The germination rate in the water control was 0%. Error bars indicate standard deviations. Different letters indicate significant differences among samples (Tukey test, *P* < 0.05, n = 3).

Various sugars and L-amino acids were used as carbon sources and separately mixed with KNO_3_ to examine their effects on the germination of resting spores. The sterile spores basically did not germinate, while there were significant differences in the germination rates of non-sterile spores (Fig 8). Germination rates of non-sterile spores were up to 56% when incubated in mixtures of carbon sources and potassium nitrate after 14 days. Carbon sources alone were not able to stimulate germination. This indicates that suitable carbon sources and nitrate are important for stimulating the germination of non-sterile spores.

## 4. Discussion

### 4.1. Role of root exudates in spore germination

Previous studies have suggested that root exudates of several host and non-host plants can stimulate resting spore germination of *P*. *brassicae* under experimental conditions [19,20]. It is interesting to note that this stimulation was not necessarily host-specific. For example, resting spores incubated with root exudates from the non-host *Lolium perenne* had higher germination rates than those with root exudates from the host plant *B*. *rapa* var. *pekinensis* [33]. A complex hexasaccharide carbohydrate derived from *B*. *oleracea* root exudates was suggested to stimulate germination of resting spores [34]. Based on these findings, we performed a series of experiments in order to confirm this proposed mechanism by root exudates in stimulating resting spore germination. A hydrophobic root exudate trapping system (HTS) was established for trapping sterile exudates from undisturbed growing roots. Similar systems have been applied in several previous studies to collect root exudates [35–37].

In a series of bioassays conducted with root exudates collected by HTS as well as by PDC, we found that the native root exudates collected with both methods were not able to stimulate the germination of sterile *P*. *brassicae* spores (S2 Table). The effects of eluates from XAD4 and XAD8 resin on stimulation of germination were similar and there was no host specificity, even though the composition of root exudates from different plant species differed. We therefore conclude that the root exudates are not the direct stimulation factor which is contrary to previous studies. This, however, does not exclude an indirect impact of root exudates on spore germination by regulating the soil microbial communities, which in turn may influence the pathogen.

It is well known that *P*. *brassicae* is an obligate biotrophic pathogen, which requires the host plant for completion of its life cycle. However, germination of resting spores in the soil and root infection by primary zoospores are two distinct processes. We hypothesize that resting spore germination may be not limited to the presence of host plants, since resting spores can germinate without plants. Moreover, formation of primary plasmodia has been also observed in roots of non-host plants, however, not allowing the pathogen to complete its life cycle [38,39]. Our data suggest that the presence of host plants is essential for pathogen propagation, but not for germination of resting spores.

### 4.2. Role of soil bacteria in spore germination

Further data gained in the present study indicated that soil microorganisms are involved in stimulating germination. The first indication derived from the observation that germination rates of resting spores incubated in non-sterile soil suspensions were significantly higher than in sterile soil suspension (Fig 1). Moreover, resting spores incubated in non-autoclaved soil had a significantly higher germination rate than in autoclaved soil at the same moisture level (Table 2). These results implied an important role of soil microbes in stimulating the germination of resting spores. Moreover, *P*. *brassicae* thus represents a unique case of lacking general suppressiveness by soil microbes on spore germination, as widely found in soils [21].

To further explore the stimulatory factors, we found that both bacterial suspension or filtrate induced germination of non-sterile spores only in the presence of Hoagland solution, while surface-sterilized resting spores remained dormant (Fig 2). This indicated that the tested bacteria strains are not able to induce germination alone and that the microorganisms in non-sterile spore suspension that originated from the surface of root galls is essential. Furthermore, we noticed that nitrate in Hoagland solution is the nutrient required to stimulate resting spore germination. However, experiments with ^15^N isotope labelled nitrate excluded a direct stimulatory effect of nitrate on resting spore germination (Fig 4). *P*. *brassicae* resting spores lack genes encoding for proteins involved in nitrate uptake [40,41] which thus excludes a direct role of nitrate to initiate the germination process.

Subsequently, through 16s rRNA gene amplicon sequencing, we found that the initial bacterial community in non-sterile spore suspension was a starting point, which is shifted to stimulatory communities by different treatments, hence inducing different spore germination rates. The treatments, bacterial suspension/filtrate and nitrate actually served as carbon and nitrogen sources to modulate the initial soil bacterial community, converting it into stimulatory communities. Our data further provided evidence (Fig 8) that instead of bacterial suspension/filtrate, various sugars and amino acids were suitable carbon sources, which stimulated the germination of non-sterile spores in the presence of nitrate. We suppose that suitable sugars and amino acids together with nitrate are able to alter the initial bacterial community to stimulatory communities, thereby the resting spores can germinate.

The present study provides evidence that a shift of bacterial communities caused by suitable carbon and nitrogen sources is crucial for stimulating the germination of resting spores. Our data indicate that the diversity and composition of bacterial communities changed after 7 days of incubation compared to the initial community in the non-sterile spore suspension (Fig 5). *Gammaproteobacteria* was the dominant class in the initial bacterial communities, but its relative abundance decreased after 7 days of incubation giving rise to *Sphingobacteriia* and *Flavobacteriia*, especially in the ‘high’ germination group.

Based on these results, we conjecture that certain soil bacteria (stimulating bacteria) exist in soil which are capable of specifically stimulating the germination of *P*. *brassicae* resting spores. In addition, we assume the existence of some other bacteria (stimulation suppressing or non-stimulating bacteria) which are competing with the previous group. This competitive relationship may be modified by suitable carbon sources and nitrate and shift soil bacterial communities from suppressive or non-stimulating to stimulating. In order to identify the stimulating bacteria, we compared the bacterial communities between ‘high’ and ‘low’ germination rate samples. As shown in the summary of Fig 6, there were clear differences in microbial profiles between the two groups. The genera *Sphingobacterium*, *Achromobacter*, *Stenotrophomonas*, *Agrobacterium*, *Chryseobacterium* and *Flavobacterium* were more abundant in the ‘high’ germination group, while genera *Chitinophaga*, *Luteibacter*, *Pseudomonas*, *Gulosibacter*, *Leifsonia*, *Rhizobium*, *Variovorax*, *Mucilaginibacter* and *Tardiphaga* prevailed in the ‘low’ germination rate group. Particularly, several abundant genera in the ‘low’ germination rate group have shown the ability to promote plant growth and contribute to protection against pathogenic bacteria, such as *Rhizobium*, *Pseudomonas* and *Luteibacter* [42–44]. Moreover, we found that ASVs related to nitrate reduction, nitrate respiration and nitrogen respiration were more enriched in the ‘high’ germination rate group. This may indicate that these nitrate metabolism related bacteria are essential for the stimulation of spore germination and explain the specific stimulating effect of nitrate on *P*. *brassicae* resting spore germination.

Further support for a role of a modulated soil microbiome in spore germination derives from significant correlations between specifically enriched genera such as *Stenotrophomonas*, *Chryseobacterium*, *Flavobacterium* and germination rates (Fig 7A). The co-occurrence network showed potential relationships among bacteria within the community (Fig 7B). Notably, *Stenotrophomonas* has been assigned to functions related to nitrate reduction, nitrate respiration and nitrogen respiration (S2 File). Some species of *Chryseobacterium* and *Flavobacterium* are also known for nitrate reduction. Besides, many species of these genera have been found in association with plants, such as oilseed rape, maize, wheat and various weeds. We therefore suppose that a soil microbiome enriched with particular bacterial taxa is involved in the stimulation of *P*. *brassicae* resting spore germination. These significantly enriched bacteria in the high germination rate group are likely to be the key factors associated with triggering spore germination. Although the contribution of individual bacterial taxa within the stimulating community is not yet clear, this study presents some potential candidate groups for future research to explore their stimulatory activity and the underlying mechanism of stimulation.

### 4.3. Integration of stimulatory factors in a ‘pathobiome’ model

Recent studies have increased awareness of a ‘pathobiome’ concept describing the interaction between pathogen, host and the associated microbial community that may influence or drive pathogenic processes [22,23]. Many studies have been devoted to exploring how a single pathogen interacts with its host, without considering the role of the overall microbial environment. The pathobiome raises the question about the composition and diversity of microbiota present in a healthy individual and the factors promoting the emergence of disease-causing pathobiomes. Understanding the interaction of microbiota with pathogens and hosts promises new insights into the nature of pathogenesis, thereby generating novel options for disease control.

A peculiarity of pathobiomes is that they involve multiple complex interactions within microbial communities, the host and the environment, and are subject to strong selection pressures [45]. Pathogens must adapt to the selective pressures associated with these complex ecosystems, specifically host defense mechanisms, competition with other microorganisms, as well as environmental variations affecting their survival and/or dissemination outside their hosts [46]. It is therefore critical to integrate ecological and evolutionary concepts of microbial communities to be able to fully comprehend the roles that ecosystem composition and structure play in the emergence of pathogens and the expression of pathogenicity.

In the present study, we investigated the influence of abiotic and biotic factors on *P*. *brassicae* resting spore germination. A bacterial community with certain characteristics was involved in triggering the germination of resting spores, which is the first step of its pathogenic processes. We suppose that the relationship between *P*. *brassicae* resting spores and its associated stimulating microbes may be one of the cases that fits to the concept of pathobiome.

**Fig 9:**
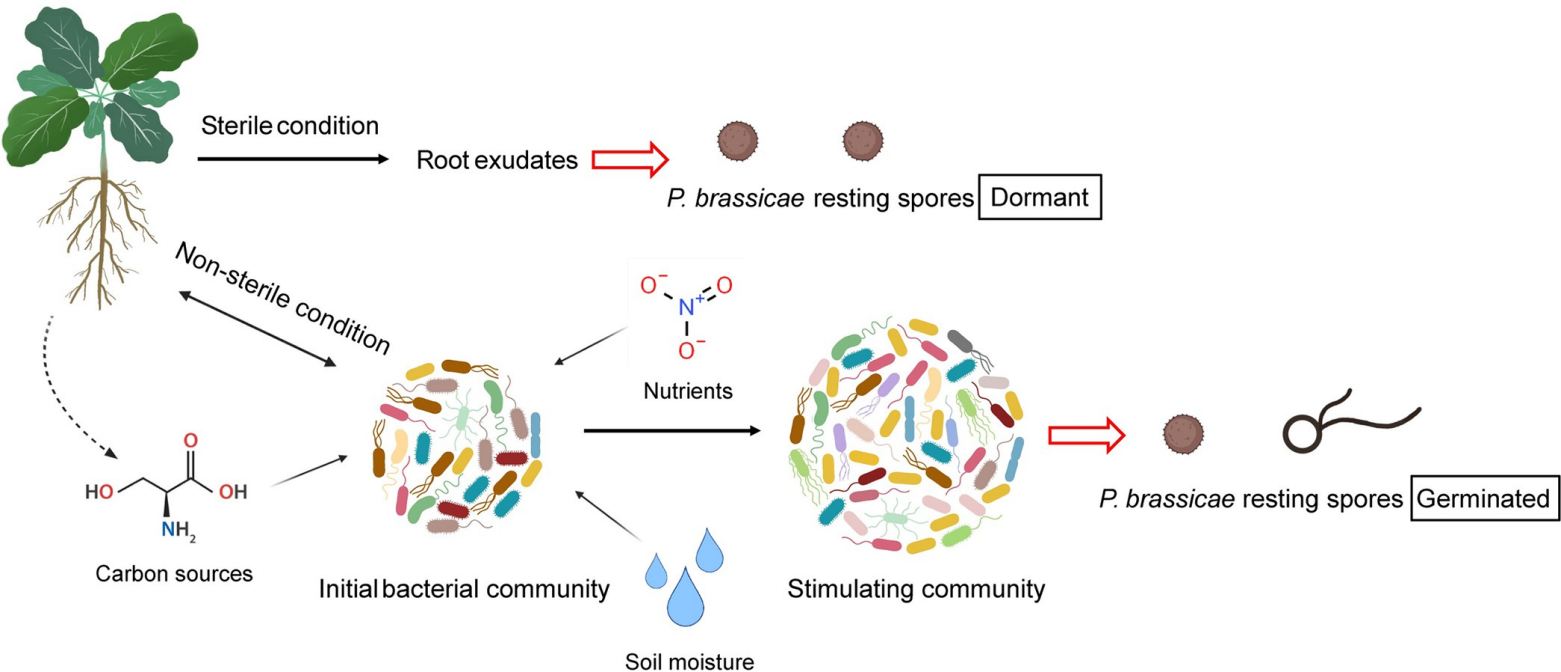
A multifactorial pathobiome model of factors affecting the germination of *Plasmodiophora brassicae* resting spores. Root exudates collected under sterile conditions cannot stimulate the germination of resting spores, while they may serve as a carbon source to modulate the microbial community under non-sterile conditions. The initial microbial community is reshaped by environmental factors (e.g. carbon sources, nutrients and soil moisture) to a community stimulating the germination of *P*. *brassicae* resting spores.

Consequently, a pathobiome model is proposed representing the putative plant-microbiome-pathogen interactions associated with breaking spore dormancy of *P*. *brassicae* in soil (Fig 9). Firstly, pure root exudates collected under sterile conditions are not the direct stimulants on resting spore germination. Secondly, the initial microbial community is modulated by environmental factors like carbon sources, nutrients and soil moisture which give rise to bacterial taxa stimulating *P*. *brassicae* spore germination and may repress non-stimulating or suppressive species. Root exudates may be one of the carbon sources modulating the soil microbial community, which have an indirect effect on the stimulation of resting spore germination. From the perspective of *P*. *brassicae*, interaction with multiple species of soil bacteria to initiate the germination process is a sophisticated strategy for long-term survival. Under natural conditions, soil bacteria communities rapidly respond to environmental changes by constantly sensing and adapting to the nutritional and physical conditions [47,48]. Therefore, the structure of bacterial communities comprehensively reflects the environmental chemical and physical conditions and represents a highly sensitive trigger for breaking dormancy under suitable conditions. Such a mechanism of triggering resting spore germination may be more reliable than a single factor like root exudates released from a plant.

Nonetheless, the presence of host plants is still essential for *P*. *brassicae* to complete its life cycle as this requires a biotrophic relationship. The ‘holobiont’ concept [49] considers plants and their associated microbiota as an entity, which is the result of evolutionary selection between plants and microorganisms and contributes to the overall stability of the system. Several studies have noted the presence of ‘core microbiota’, a subset of microbial lineages which are reproducibly associated with a particular host plant in almost all the communities [50–52]. We speculate that key stimulation bacteria which are frequently found in the rhizosphere may be one of the core microbiota regulated by the root exudates of *P*. *brassicae* host plants and may thus help the pathogen to recognize its host in the soil.

It is interesting to note that nitrate is a more favorable nitrogen source than ammonium to form germination-stimulatory bacterial communities. Nitrate is the most prevalent reactive form of nitrogen required by plants, although ammonium and amino acids are also present [53,54]. Plants may steer the formation and growth of lateral roots into NO_3_^–^ rich patches in the soil [55,56]. Moreover, nitrate is abundant in the soil solution and moves with runoff water, while ammonium ions are attached to the soil cation exchange complex which limits its mobility. We suppose that these characteristics of nitrate in the presence of stimulating bacteria and sufficient soil moisture may be beneficial for *P*. *brassicae* in guiding it towards plant roots.

A better understanding of these complex interactions provides us with novel directions for optimizing integrated control strategies of clubroot disease. The present results elucidate the importance of microbial communities in the germination of *P*. *brassicae* resting spores and indicate the presence of intense microbe-microbe competition. Synthetic communities (SynComs), artificially manipulated for specific purposes [57], could be introduced in soil as a tool to control clubroot disease. For example, application of synthetic communities consisting of stimulation-associated bacteria before sowing or in the absence of susceptible host plants may be capable of triggering the germination of resting spores thereby reducing the soil inoculum. SynComs of microorganisms with broad, persistent and durable plant-growth-promoting traits may suppress stimulation-associated bacteria and enhance plant resistance thereby alleviating disease severity [58,59].

Altogether, the present study revises former views and presents an entirely novel understanding of the clubroot pathobiome highlighting a significant role of soil microbiota and their modulation by additional chemical and physical soil factors. The provided novel insights into this complex system may enable the development of innovative tools for integrated sustainable control of this notorious pathogen.

## Supporting information

S1 Fig A hydrophobic root exudate trapping system (HTS) based on perlite substrate to collect root exudates under sterile conditions. The root exudates (red arrows) released from undisturbed living roots are selectively captured by the columns containing XAD8 and XAD4 resin. Hoagland solution (blue arrows) is continuously circulated through the entire system to sustain plant growth. The container is covered with a plastic membrane with micro-porous filter strips allowing gas exchange under sterile conditions (right).
(TIF)

S2 Fig Germinated and non-germinated spores in soil samples as observed under the microscope. Germinated spores (GS, red arrows) and non-germinated spores (NS, black arrows) in soil samples as observed under the microscope.
(TIF)

S1 Table Inorganic compounds used in spore germination bioassays. 1) Concentration of inorganic compounds used for bioassays based on full strength or 1/10 strength Hoagland solution in the product formulation.
(DOCX)

S2 Table Germination rates (G%) of sterile spores incubated with root exudates collected from PDC and HTS. (DOCX)

S3 Table Germination rates (G%) of sterile resting spores of *P*. *brassicae* incubated with different bacterial suspensions or filtrate with Hoagland solution or sdH_2_O after 7 days. (DOCX)

S4 Table Co-occurrence network properties of the bacterial communities in the high and low germination rate groups. (DOCX)

S1 File Number of reads for the individual samples, abundance table and taxonomy annotation. (XLSX)

S2 File Report of bacterial functional annotations using FAPROTAX. (XLSX)
