# Supplementary figures and images for: The soil bacterial community regulates germination of Plasmodiophora brassicae resting spores rather than root exudates
Source: PLoS Pathog. 2023 Mar 2;19(3):e1011175. doi: 10.1371/journal.ppat.1011175 (PMC9980788; doi:10.1371/journal.ppat.1011175)

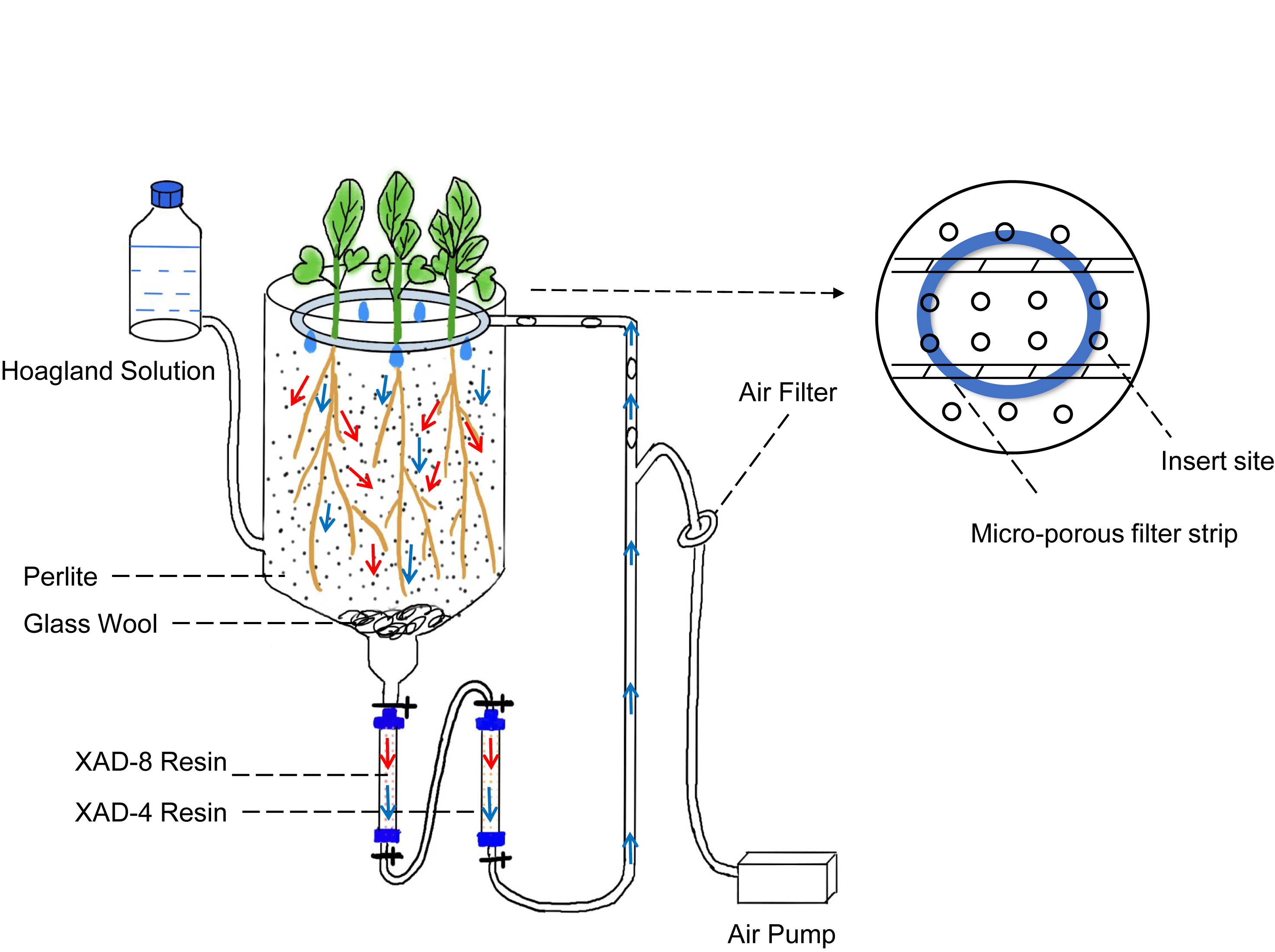

Supplement: S1 Fig — The root exudates (red arrows) released from undisturbed living roots are selectively captured by the columns containing XAD8 and XAD4 resin. Hoagland solution (blue arrows) is continuously circulated through the entire system to sustain plant growth. The container is covered with a plastic membrane with micro-porous filter strips allowing gas exchange under sterile conditions (right). (TIF) [file ppat.1011175.s001.tif]

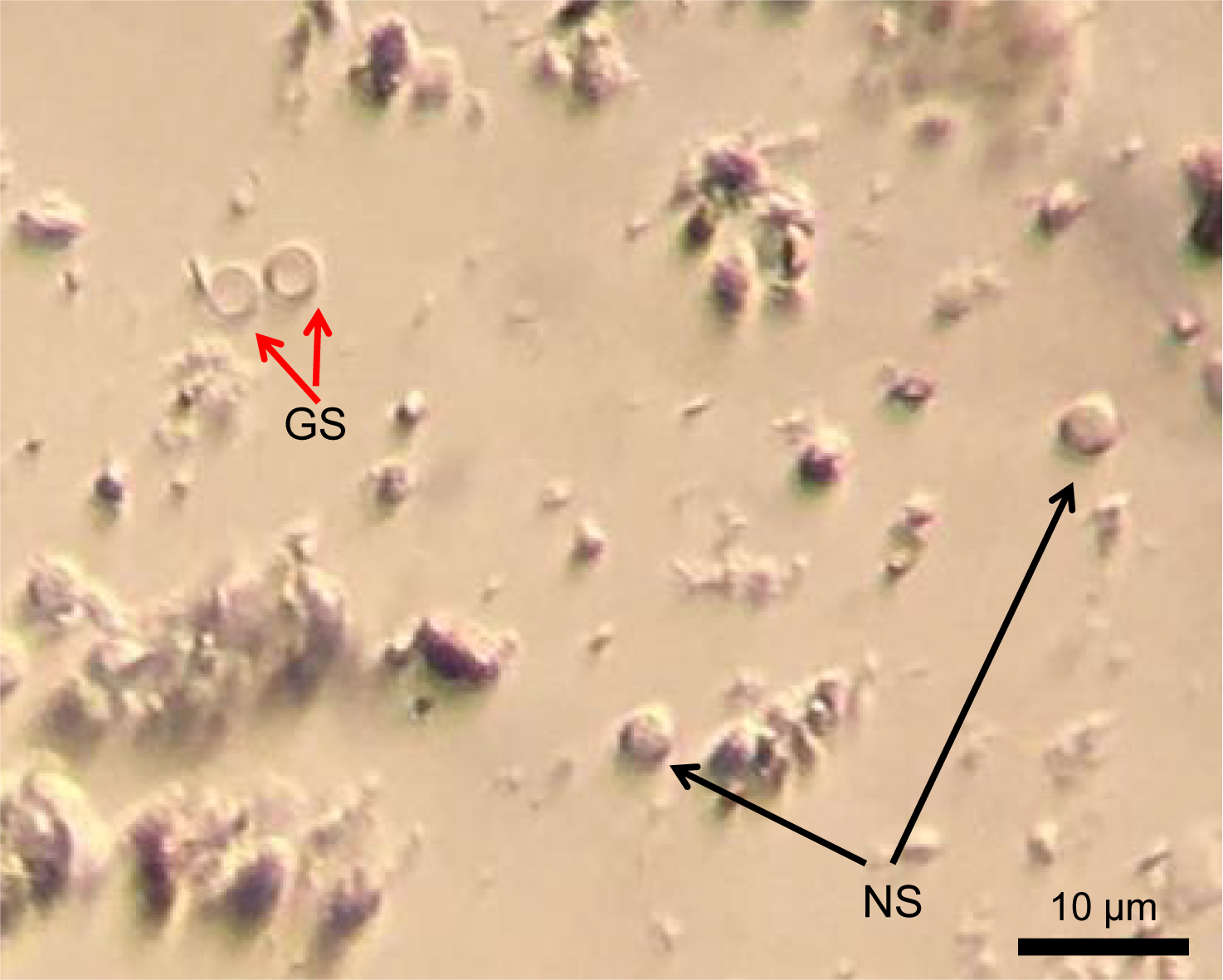

Supplement: S2 Fig — Germinated spores (GS, red arrows) and non-germinated spores (NS, black arrows) in soil samples as observed under the microscope. (TIF) [file ppat.1011175.s002.tif]
