# Supplementary material for: The soil bacterial community regulates germination of Plasmodiophora brassicae resting spores rather than root exudates
Source: PLoS Pathog. 2023 Mar 2;19(3):e1011175. doi: 10.1371/journal.ppat.1011175 (PMC9980788; doi:10.1371/journal.ppat.1011175)
Supplement: S1 Table — 1) Concentration of inorganic compounds used for bioassays based on full strength or 1/10 strength Hoagland solution in the product formulation. (DOCX) [file ppat.1011175.s003.docx]

**S1 Table.** Inorganic compounds used in spore germination bioassays

| **Chemical** | **Manufacturer** | **Applied Conc.^1^ (mg/l)** |
| --- | --- | --- |
| Ca(NO_3_)_2_ | Merck KGaA, GER | 656.40/65.64 |
| KNO_3_ | Carl Roth GmbH & Co. KG, GER | 606.60/60.66 |
| MgSO_4_ | Carl Roth, Germany | 240.76/24.076 |
| NH_4_H_2_PO4 | Sigma-Aldrich, Germany | 115.03/11.503 |
| H_3_BO_3_ | Carl Roth, Germany | 2.86/0.286 |
| CuSO_4_·5H_2_O | Carl Roth GmbH & Co. KG, GER | 0.08/0.008 |
| ZnSO_4_·7H_2_O | Merck KGaA, GER | 0.22/0.022 |

1. Concentration of inorganic compounds used for bioassays was based on full strength or 1/10 strength Hoagland solution in the product formulation.
