# Supplementary material for: The soil bacterial community regulates germination of Plasmodiophora brassicae resting spores rather than root exudates
Source: PLoS Pathog. 2023 Mar 2;19(3):e1011175. doi: 10.1371/journal.ppat.1011175 (PMC9980788; doi:10.1371/journal.ppat.1011175)
Supplement: S2 Table — (DOCX) [file ppat.1011175.s004.docx]

**S2 Table.** Germination rates (G%) of sterile spores incubated with root exudates collected from PDC and HTS

|  | Petri dish cultivation (PDC) | |
| --- | --- | --- |
|  | Hoagland (G%) | sdH_2_O (G%) |
| Oilseed rape | 1.00±1.41 | 0.25±0.50 |
| Ryegrass | 1.00±1.15 | 0.75±0.96 |
| Tomato | 1.25±0.50 | 0.25±0.50 |
| Blank | 0.75±0.96 | 0.33±0.58 |

(Value = mean ± SD, n=4)

|  | | Hydrophobic trapping system (HTS) | |
| --- | --- | --- | --- |
|  | | Hoagland (G%) | sdH_2_O (G%) |
| **Oilseed rape** | |  |  |
| XAD4 | BBCH14 | 1.67±1.21 | 0.33±0.52 |
|  | BBCH52 | 2.50±2.17 | 0.67±0.82 |
| XAD8 | BBCH14 | 2.17±1.83 | 0.33±0.52 |
|  | BBCH52 | 1.33±1.03 | 0.67±1.21 |
| **Ryegrass** | |  |  |
| XAD4 | BBCH14 | 3.50±2.35 | 0.33±0.82 |
|  | BBCH52 | 3.17±1.17 | 0.33±0.52 |
| XAD8 | BBCH14 | 1.83±1.72 | 0.50±0.55 |
|  | BBCH52 | 2.00±2.68 | 0.67±1.21 |
| **Tomato** | |  |  |
| XAD4 | BBCH14 | 0.83±0.75 | 0.50±0.84 |
|  | BBCH52 | 2.33±1.75 | 0.67±0.82 |
| XAD8 | BBCH14 | 0.83±0.98 | 0.50±0.55 |
|  | BBCH52 | 1.00±1.26 | 0.83±0.98 |
| **Blank** | |  |  |
| XAD4 | BBCH14 | 2.50±2.07 | 0.17±0.41 |
|  | BBCH52 | 3.17±2.14 | 0.33±0.82 |
| XAD8 | BBCH14 | 1.00±0.63 | 0.50±0.55 |
|  | BBCH52 | 1.67±0.63 | 0.33±0.82 |

(Value = mean ± SD, n=6)
