# Supplementary material for: The soil bacterial community regulates germination of Plasmodiophora brassicae resting spores rather than root exudates
Source: PLoS Pathog. 2023 Mar 2;19(3):e1011175. doi: 10.1371/journal.ppat.1011175 (PMC9980788; doi:10.1371/journal.ppat.1011175)
Supplement: S3 Table — (DOCX) [file ppat.1011175.s005.docx]

**S3 S3 Table.** Germination rates (G%) of sterile resting spores of *P. brassicae* incubated with different bacterial suspensions or filtrate with Hoagland solution or sdH_2_O after 7 days.

| **Strain** | **Treatment** | **Hoagland (G%)** | **sdH_2_O (G%)** |
| --- | --- | --- | --- |
| A4 | filtrate | 1.67±1.53 | 1.00±1.00 |
|  | suspension | 2.33±1.53 | 0.33±0.58 |
| *B. subtilis* | filtrate | 2.67±1.53 | 0.67±1.15 |
|  | suspension | 2.33±2.08 | 0.33±0.58 |
| *E. coli* | filtrate | 2.33±1.53 | 0.33±0.58 |
|  | suspension | 2.67±1.53 | 0.33±0.58 |
| Iso4 | filtrate | 1.67±2.08 | 1.33±1.53 |
|  | suspension | 2.33±0.58 | 0.67±0.58 |

(Value = mean ± SD, n=3)
