# Supplementary material for: The soil bacterial community regulates germination of Plasmodiophora brassicae resting spores rather than root exudates
Source: PLoS Pathog. 2023 Mar 2;19(3):e1011175. doi: 10.1371/journal.ppat.1011175 (PMC9980788; doi:10.1371/journal.ppat.1011175)
Supplement: S4 Table — (DOCX) [file ppat.1011175.s006.docx]

| **Network properties** | **High germination rate group** | **Low germination rate group** |
| --- | --- | --- |
| **Observed networks** |  |  |
| Number of nodes | 60 | 34 |
| Number of edges | 172 | 38 |
| Clustering coefficient | 0.762162 | 0.780822 |
| Number of clusters | 6 | 9 |
| Average path length | 3.164414 | 1.508197 |
| **Random Networks** |  |  |
| Clustering coefficient | 0.0966 ± 0.0151 | 0.0642 ± 0.0442 |
| Number of clusters | 1.1400 ± 0.3722 | 4.5760 ± 1.4340 |
| Average path length | 2.4963 ± 0.0253 | 3.7045 ± 0.4250 |

**S4 Table.** Co-occurrence network properties of the bacterial communities in the high and low germination rate groups
